# Functional Characterization of a Female-Biased Chemoreceptor of the Codling Moth (*Cydia pomonella*) Responding to Aldehydes and Other Volatile Compounds

**DOI:** 10.1007/s10886-025-01579-1

**Published:** 2025-02-25

**Authors:** Alberto Maria Cattaneo, Charles A. Kwadha, Heidi Pullmann-Lindsley, Anna L. Erdei, R. Jason Pitts, William B. Walker

**Affiliations:** 1 Department of Plant Protection Biology, Chemical Ecology. Lomma - Campus Alnarp, Swedish University of Agricultural Sciences, 234 56 Alnarp, Sweden; 2 Present Address: Department of Entomology and Plant Pathology, North Carolina State University, Raleigh, NC 27695-7616 USA; 3 Department of Biology, Baylor University, 101 Bagby Avenue, Waco, TX 76706 USA; 4 Present Address: Department of Biological and Molecular Sciences, George Fox University, 414 N Meridian Street, Newberg, OR 97132 USA; 5 USDA-ARS, Temperate Tree Fruit and Vegetable Research Unit, 5230 Konnowac Pass Road, Wapato, WA 98951 USA

**Keywords:** *Cydia pomonella* chemoreceptors, Heterologous expression, Transgenic *Drosophila melanogaster*, Single sensillum recording (SSR), Gas-chromatography-coupled SSR (GC-SSR), *Xenopus* oocytes

## Abstract

With the advent of semiochemical-based control strategies used to mitigate damage of agricultural pest moths, many studies have focused on the function of male-specific putative pheromone receptors (PRs). In this investigation, we instead isolated, heterologously expressed, and functionally characterized a female-biased candidate PR, CpomOR22, from the codling moth, *Cydia pomonella*. Using transgenic *Drosophila melanogaster* for single sensillum recording (SSR) and gas-chromatographic SSR, we tested both synthetic ligands and various apple headspace extracts, identifying saturated and unsaturated aldehydes (nonanal, decanal, undecanal, dodecanal; (*Z*)-4-undecenal and (*Z*)-6-undecenal) among the most active ligands. Parallel experiments expressing CpomOR22 in *Xenopus* oocytes confirmed the binding of nonanal, decanal and undecanal and revealed lactones (γ-undecalactone and δ-dodecalactone) and several carboxylic acids as additional active compounds. The renowned ecological importance of aldehydes for the codling moth and the potential for newly identified ligands, such as lactones, may inform innovative control strategies based on novel semiochemicals to interfere with the female-specific chemosensory systems of this insect.

**Supplementary Information:**

The online version contains supplementary material available at 10.1007/s10886-025-01579-1.

## Introduction

The codling moth, *Cydia pomonella* (Lepidoptera: Tortricidae), is a carpophagous global pest of apple, pear, and walnuts. For decades, sustainable methods like male-mating disruption (Witzgall et al. 2008; 2010), and other alternatives based on attract-and-kill (Charmillot et al. 2000; Krupke et al. 2002), have been successful for the management of this pest. Despite this, there is an ongoing and growing concern that these techniques may no longer reduce the damage below economically acceptable thresholds, given their high costs, rendering them less competitive when compared with the most common applications of insecticides (Miller and Gut 2015; Carde and Minks 1995; Gregg et al. 2018). While the future of mating disruption will require area-wide management schemes (Carde and Minsk 1995; McGhee et al. 2011) and expanded collaborations between fundamental and applied scientists, as well as a meaningful inclusion of industrial partners (Miller and Gut 2015), costs and delays associated with registration, and harmonization of the regulatory requirements among different countries, can represent a serious obstacle in the development of sprayable attract-and-kill products (Gregg et al. 2018).

At the beginning of this millennium, a ripe pear-released volatile, ethyl (*E,Z*)−2,4-decadienoate, commonly known as pear ester (Light et al. 2001), was identified to evoke olfactory neuronal responses in both male and female *C. pomonella* (Ansebo et al. 2004, 2005). Incorporating this ligand into codlemone baited traps enabled monitoring of moth flight patterns in orchards but trapping varied across seasons and crops (Landolt et al. 2007; Light et al. 2001; Kutinkova et al. 2005; Thwaite et al. 2004; Knight et al. 2012). Whether kairomones, like pear ester, may modulate food-finding, oviposition site seeking, or both, remains inconclusive. Gravid females of *C. pomonella* use host kairomones, for example from apple, to locate and select a suitable oviposition site (Landolt et al. 2007, Landolt and Guédot 2008; Preti et al. 2021; Hern and Dorn 2004). In addition, studies that are more dated showed that mated females of *C. pomonella* prefer apples infested with conspecific larvae over uninfested apples (Reed and Landolt 2002; Yan et al. 1999; Hern and Dorn 2002), suggesting that the quality of kairomones changes with arthropod infestation, subsequently impacting pest behaviour. Independently from its possible role as a semiochemical, effects from pear ester are compelling: while it is male active, and it may contribute to the disruption of male insects, it may also target females to be combined with pheromones in baited traps interfering with both sexes (Knight et al. 2022; Landolt et al. 2007). Indeed, strong bisexual kairomonal attractants for key crop pests such as the codling moth, may increase opportunities to develop attract-and-kill products that, apart from males, may target the removal of females of this species from the orchards (Gregg et al. 2018). Since the discovery of the sex pheromone bombykol in the silk moth *Bombyx mori* (Butenandt et al. 1959), recent advancements in transcriptomics and bioinformatics have enhanced the understanding of the molecular mechanisms behind chemosensory modalities among the various species belonging to the broad taxonomic group of Lepidoptera (Walker et al. 2023, 2019; Crava et al. 2022; Poivet et al. 2013; Montagné et al. 2021).

While the application of sex pheromones to disrupt males has led to more emphasis on male-specific olfactory receptors (ORs) than on the female-biased ones (Bastin-Heline et al. 2019; Löfstedt et al. 2016), few studies have deciphered female-specific ORs (Wanner et al. 2007; Anderson et al. 2009; Tom et al. 2022). For example, in *B. mori,* antennal expression of OR-proteins with a female bias is evident for several of their subunits, including BmorOR19, OR30, and ORs 45 to 48 (Wanner et al. 2007). Subsequent functional characterization showed that BmorOR19 and BmorOR45, together with BmorOR47, respond to mulberry leaf volatile components linalool and benzoic acid, respectively (Anderson et al. 2009; Pophof 1997; Heinbockel and Kaissling 1996). In the hawkmoth *Manduca sexta,* MsexOR6 and MsexOR15 are female-specific ORs, with the former expressed in the antennae and legs and the latter exclusively in the antennae. Additionally, ORs 5, 17, 26, 33, 40, and 87 exhibit biased expression in female antennae (Tom et al. 2022; Koenig et al. 2015). However, the female-biased *MsexORs* have yet to be functionally characterized.

In the codling moth, antennal transcriptome analysis comparing adults and larvae unveiled four female-specific/biased ORs: CpomOR21, CpomOR22 (also named OR15a*:* accession number CPOM22347.t1 (Wan et al. 2019)), CpomOR30, and CpomOR41 (Walker et al. 2016). Among these, CpomOR21 and CpomOR22 cluster within the traditional lepidopteran pheromone receptor (PR) clade (Krieger et al. 2004; Zhang and Löfstedt 2015). While, to date, ligands for these ORs remain unknown, it is expected that such female-biased or enriched ORs may bind to kairomones. For example, within the PR-clade of the codling moth, an OR which is highly expressed by both males and females, CpomOR3, responds to the pear ester kairomone and to its methyl-analogue methyl-(*E*,*Z*)−2,4 decadienoate (Bengtsson et al. 2014; Cattaneo et al. 2017). According to functional characterization methods based on *Xenopus* oocytes, CpomOR3 also responds, to a lesser extent, to the codling moth sex pheromone codlemone, (*E*,*E*)−8,10-dodecadien-1-ol (Wan et al. 2019), however, this finding has not been confirmed by expressing the same subunit in other heterologous methods (Cattaneo et al. 2017). Otherwise, only one codling moth PR has been functionally characterized up to now: CpomOR6a, which was reported to respond to various acetates renowned among the female-produced sex pheromone components, including (*E*,*E*)−8,10-dodecadien-1-yl acetate (Cattaneo 2018; Cattaneo et al. 2017). For *CpomOR22* specifically, putative orthologs exist, based on phylogenetic relatedness, in other tortricidae, including *Ctenopseustis obliquana, Ctenospeustis herana*, and *Epiphyas postvittana* (Walker et al. 2016; Corcoran et al. 2015; Steinwender et al. 2015), as well as other non-tortricid lepidopterans such as *B. mori* and *M. sexta* (Supplementary Fig. 1). Starting from these lines of evidence, we focused on this subunit, hypothesizing its possible involvement in perceiving signals that mediate female specific behaviors, such as oviposition, to lead our efforts in attempting its functional characterization.

To test a possible involvement of the female-biased CpomOR22 in host volatile sensing, we heterologously expressed this subunit in the *Drosophila* empty neuron system and in *Xenopus* oocytes (Gonzalez et al. 2016; Fleischer et al. 2018), after using qRT-PCR to confirm enriched expression of *CpomOR22* in female antennae. Our results illustrate that *CpomOR22* provides an olfactory channel for perceiving host-associated cues. Additionally, through assay with structural analogues, we reveal novel compounds previously not reported in the chemosensory ecology of *C. pomonella.* Altogether, the current study advances our understanding of the mechanisms of olfactory detection in *C. pomonella* females and provides a new perspective for ligand postulation in insect pests.

## Material and Methods

### Quantitative RT-PCR (qRT-PCR)

For each biological sample, antennal pairs from 100 unmated male or unmated female *C. pomonella* adults were dissected into RNA-Later. Total RNA from all samples was extracted and purified with the RNeasy Plus Mini Kit (Qiagen, Venlo, Netherlands), and cDNA was synthesized with the QuantiTech Reverse Transcription Kit cDNA synthesis kit (Qiagen) according to the manufacturer's protocol. qRT-PCR experiments were carried out with Roche Light Cycler 480 II thermocycler (Roche, Basel, Switzerland). qRT-PCR primers for each gene were designed with the IDT RealTime PCR tool (Integrated DNA Technologies; https://www.idtdna.com/scitools/Applications/RealTimePCR/) (Supplementary Table 1). For each primer assay, amplification was performed on biological triplicates of unmated male and female antennal samples with technical duplicates. For all reactions, the following contents were added: 2.0 μL of cDNA sample, 12.5 μL of enzymatic mix, iTaq SYBR Green supermix with ROX (Bio-Rad Laboratories, Hercules, CA, USA), 5.5 μL water, and 5.0 μL of gene-specific primers (100 nM final concentration of each primer), for a final reaction volume of 25 μL. The qRT-PCR amplification protocol was run as follows: initiation phase: 3 min at 95 °C; amplification phase (40 cycles): 10 s at 95 °C, 40 s at 56 °C; melting curve phase: 40–95 °C gradient, with analysis every 1.0 °C; melting curves were analyzed to verify the specificity of amplification products. Primer efficiencies were calculated for all primer pairs using a Topo4 plasmid (Invitrogen) with an insert of cloned amplicon using the same primers for the qRT-PCR assay, with six serial dilutions ranging from 2.0 ng to 2e-5 ng and a dilution factor of 10. For *OR22*, relative expression was normalized to the expression of two reference genes, *ActR2* and *HSP40*; these reference genes were selected from a pool of six candidate reference genes (Lü et al. 2018) based upon optimal stability of expression across all biological samples and primer efficiency values as close as possible to 100%. Candidate reference gene sequences were identified in our previously published transcriptome (Walker et al. 2016) or the genome database associated with the published *C. pomonella* genome (Wan et al. 2019). Relative gene expression differences across male and female samples were assessed by determination of relative quantities (primer efficiency^∆(CT)) of the *OR22* gene relative to the geometric mean of the relative quantities of the two reference genes (Vandesompele et al. 2002). ∆(CT) values for each biological replicate were calculated relative to the average CT value of the three male antennal samples (Supplementary Data File 1). To control for potential differences in olfactory gene expression in male versus female antennae, *OR22* expression differences were further compared to the expression of the odorant receptor coreceptor, *CpomOrco*. Statistical differences were assessed across conditions on binary log-transformed relative quantities using an independent t-test with two-tailed distribution and two samples with equal variance; significance was assessed at (*P* < 0.05).

### Cloning and Heterologous Expression of *CpomOR22* in *Drosophila* Empty Neuron System

A synthetic construct of the *OR22* ORF of *C. pomonella* was obtained (Eurofins Genomics, Ebersberg, Germany) as a plasmid insert in pCR2.1-Topo; the ORF was based upon its sequence identified in the antennal transcriptome (Walker et al. 2016) and was codon optimized for expression in *Drosophila melanogaster* (Supplementary Data File 2). The complete ORF encoding *CpomOR22* was amplified by PCR combining specific codon-optimized CDS-primers (Fw: 5'-ATGAAGTTTGAAGAGGCCGAC-3'; Rv: 5´-TTACTCGATAGAGGATTTGAGCATGA-3') with the pCR2.1 plasmid as a template. Purified PCR products were then cloned into the PCR8/GW/TOPO plasmid (Invitrogen, Waltham, MA, USA). Insert integrity and orientation were confirmed by Sanger sequencing, with a 3730xl DNA Analyzer (Eurofins Genomics). Cassettes with inserts were then transferred from their PCR8/GW/TOPO plasmids to the destination vector (pUASg-HA.attB, constructed by E. Furger and J. Bischof, kindly provided by the Basler group, Zürich) using the Gateway LR Clonase II kit (Invitrogen). Insert integrity and orientation were again checked by Sanger sequencing.

Transformant *D. melanogaster* lines with *pUAS-CpomOR22* were generated by Best Gene (Chino Hills, CA, USA), injecting into Best Gene Strain #24749 with genotype M{3xP3-RFP.attP}ZH-86Fb and insertion locus on the third chromosome. Crossings were performed with standard balancer lines and the *Δhalo* chromosomal background to drive the expression of *CpomOR22* in the A neuron of ab3 basiconic sensilla (ab3A OSNs) according to procedures already established in our labs (Gonzalez et al. 2016). The final crossing was performed with *w;Δhalo/CyO;pOr22a-Gal4* mutant line (Dobritsa et al. 2003; Hallem et al. 2004), and selection of *∆halo* homozygotes was based on the straight wings phenotype. The final strain tested by SSR and GC-SSR had the following genotype: *w;Δhalo;pUAS-CpomOR22/pOR22a-Gal4.* Insects were reared in our facilities at room temperature (25 ± 2 °C) on a sugar-yeast-cornmeal diet (https://bdsc.indiana.edu/information/recipes/bloomfood.html) at a relative humidity of 50 ± 5% and under 12:12 light:dark photoperiod.

### Volatile Collection From Apple Headspace

Volatile collections from *Hoplocampa testudinea* (Hymenoptera:Tenthredinidae) infested branches of apple trees (*M. domestica* v. 'Discovery', with 10–15 pieces of 1–3 cm diameter apples and 10–20 leaves, sample names abbreviated as Hoplomalus) and branches with non-infested fruits (sample names abbreviated as Malus) were used in the GC-SSR recordings. The *H. testudinea* insect-infested apples were selected to qualitatively broaden the chemical profile for screening purposes. The volatile collections were done in the laboratory using Porapak Q filled volatile collection traps. The biological samples were placed in 25 × 38 cm polyester oven roasting bags (Look, Teri- nex Ltd., England), and charcoal-filtered air was pumped into the bag at 410 mL/min to keep the bag inflated during sampling. The volatile collection traps were connected to the oven bag and fit with a Teflon tube, while the headspace was drawn through each trap at the rate of 200 mL/min for 2 h 40 min, 6 h, 14 h, or 20 h depending on the sample (2 h 40 min: Malus 559, Hoplomalus 560, Malus 561; 6 h: Hoplomalus 583, Malus 590;14 h: Hoplomalus 566, Malus 603; 20 h Hoplomalus 500). The choice of this method was based on the evidence that the content of volatiles from both infested and non-infested apples may be very diverse across time, both qualitatively and quantitatively (Hern and Dorn 2001). We opted for this approach to attempt to increase the likelihood of enriching headspace with a more representative set of volatiles. The samples were desorbed from the volatile traps with 200–400 uL hexane, and 1.0 ug heptyl acetate was added as an internal standard to the volatile extracts. The extracts were stored at −40 °C in melted glass capillaries. The choice of using hexane as solvent came from our previous protocols (Cattaneo et al. 2023; Pettersson and Cattaneo 2024) based on the reported evidence of gaining higher sensitivity for moth ORs heterologously expressed in *Drosophila* OSNs, when odors are diluted in this solvent rather than others, like paraffin oil or methylene dichloride (Wang et al. 2016).

### Single Sensillum Recordings

*CpomOR22* expressed in the A neuron of ab3 basiconic sensilla was tested through single sensillum recordings (SSR), adapting the protocols we recently described (Cattaneo et al. 2023). In brief, three- to eight-day-old female flies were immobilized in 100 μL pipette tips with only the top half of the head protruding. The right antenna of each insect was gently pushed with a glass capillary against a piece of glass. This piece of glass and the pipette tip were fixed with dental wax on a microscope slide. Electrolytically sharpened tungsten electrodes (Harvard Apparatus Ltd, Edenbridge, United Kingdom) were used to penetrate the insect's body: the reference electrode was manually inserted in the right eye of the fly, while the recording electrode was maneuvered with a DC-3 K micromanipulator equipped with a PM-10 piezo translator (Märzhäuser Wetzler GmbH, Wetzler, Germany) and inserted in ab3-sensilla. Signals coming from the olfactory sensory neurons were amplified ten times with a probe (INR-02, Syntech, Hilversum, the Netherlands), digitally converted through an IDAC-4-USB (Syntech) interface, and visualized and analyzed with the software Autospike v. 3.4 (Syntech). To carry the odorant stimulus, prevent antennal dryness, and minimize the influence of background odors from the environment, a constant humidified flow of 2.5 L/min charcoal-filtered air was delivered through a glass tube and directed to the preparation. To confirm the expression of *CpomOR22*-transgenes, basic spiking of ab3-neurons was compared with parental flies *Δhalo-*homozygous (*w;Δhalo;pOr22a-Gal4* and *w; Δhalo;* + mutants) (Cattaneo et al. 2023). A panel of 27 odorants (Table 1) was made of synthetic compounds from our collection (Cattaneo et al. 2023; Lebreton et al. 2017), including ligands that have been previously reported among codling moth pheromones, compounds emitted from fruit and yeast (Bengtsson et al. 2014, 2001; Bäckman et al. 2000; Witzgall et al. 2012; Jennings and Sevenants 1964), and a few novel ligands. Among these, we included some primary pheromones and kairomones of *C. pomonella* based on previous functional studies on its primary pheromone receptors (Bengtsson et al. 2014; Cattaneo et al. 2017). In the screening, we also added (*Z*,*Z*)−3,13-Octadecadien-1-yl acetate (CAS: 53,120–27-7), a main pheromone compound from the red-belted clearwing moth *Synanthedon myophaeformis*, for which further investigation will be part of a separate study.

**Table 1:** Compounds screened by SSR on *D. melanogaster* expressing CpomOR22. Compound classes, compound purity, source, CAS numbers, and physicochemical properties are reported, including averages and standard deviation of the response to each ligand, based on five replicates. The table reports average and standard error from raw spiking (∆spks/0.5 s) and from the respective values, upon normalization on the average from the single replicate, as done in Cattaneo et al. (2023). Sign.: asterisks denote significant effects (Wilcoxon Signed Rank Test, *p* < 0.05, Supplementary Data File 3). Sources, from which the compounds have been initially found, and the respective studies, are indicated in the table. Letter on species source denotes references as it follows: ^A^Bäckman et al. 2000; ^B^Bengtsson et al. 2001; ^C^Cattaneo et al. 2023; ^D^Hern and Dorn 2004; ^E^Lebreton et al. 2017; ^F^Witzgall et al. 2012

Compound class	Compound name	Source	CAS	Purity (%)	Mol. weight (g/mol)	Vapor pressure (mmHg @ 25 °C)	Raw spiking		Normalized		*p*-val	Sign
							**Average**	**St.Er**	**Average**	**St.Er**		
Terpene alcohol	**(R)-linalool**	*Malus domestica* cv. Discovery^A^	126–91-0	97	154.2527	0.09100	−1.60	5.04	−0.79	0.77	0.465	
Terpene alcohol	**(S)-linalool**	*Malus domestica* cv. Discovery^A^	78–70-6	97	154.2527	0.01600	5.00	5.10	−0.23	0.75	0.345	
Aromatic alcohol	**2-phenylethanol**	*Metschnikowia* ssp^B^	8/12/1960	99	122.1669	0.08680	3.20	6.89	−0.54	0.86	0.715	
Aliphatic alcohol	**3-octanol**	Our collection^C^	589–98-0	95	130.2307	0.51200	1.40	6.19	−0.15	0.48	0.588	
Aliphatic alcohol	**(*E*,*E*)-alpha-farnesol**	*Malus domestica* Cv. Granny Smith^D^	106–28-5	95	222.3714	0.00037	4.20	4.42	−0.10	0.52	**0.043**	*
Aliphatic alcohol	**(*Z*)−3-hexenol**	*Malus domestica* cv. Discovery^A^	928–96-1	98	100.1608	1.03900	5.40	4.75	−0.13	0.76	0.345	
Aliphatic alcohol	**(*E*,*E*)−8,10-dodecadien-1-ol**	*C. pomonella*^E^	33,956–49-9	89	182.3065	0.00100	4.40	5.16	−0.29	0.80	0.500	
Saturated aldehyde	**nonanal**	*Malus domestica* cv. Discovery^A^; *Metschnikowia* ssp^B^	124–19-6	95	142.2417	0.53200	92.60	8.45	9.30	2.53	**0.043**	*
Monoinsaturated aldehyde	**(*Z*)−4-undecenal**	Our collection^C^	68,820–32-6	90	168.2796	0.04500	62.80	12.21	5.78	1.23	**0.043**	*
Monoinsaturated aldehyde	**(*Z*)−6-undecenal**	Novel	60,671–73-0	95	168.2796	0.04540	93.00	14.25	8.96	2.15	**0.043**	*
Polyunsaturated aldehyde	**(*E*,*E*)−2,4-decadienal**	Our collection^C^	25,152–84-5	89	152.2367	0.03000	14.60	3.78	1.31	0.42	**0.043**	*
Terpenoid aldehyde	**citral**	Our collection^C^	5392–40-5	95	152.2367	0.20000	6.80	7.12	−0.36	1.17	0.500	
Terpenoid aldehyde	**β-cyclocitral**	Our collection^C^	432–25-7	78	152.2370	0.17600	−1.80	2.08	−0.36	0.40	0.500	
Aliphatic ketone	**2-heptanone**	*Metschnikowia* ssp^B^	110–43-0	98	114.1878	4.73200	−6.00	4.80	−0.95	0.58	0.345	
Fatty acid ester	**ethyl acetate**	Our collection^C^	141–78-6	99.5	88.1062	111.71600	−5.40	4.81	−0.96	0.59	0.225	
Fatty acid ester	**ethyl hexanoate**	*Malus domestica* Cv. Granny Smith^D^	123–66-0	98	144.2139	1.66500	8.60	6.89	0.04	0.83	0.225	
Fatty acid ester	**ethyl-(*E*,*Z*)−2,4-decadienoate**	*Pyrus communis* Cv. Williams^F^	3025–30-7	85	196.2898	0.01000	7.40	4.46	0.15	0.51	**0.043**	*
Fatty acid ester	**methyl-(*E*,*Z*)−2,4-decadienoate**	*Pyrus communis* Cv. Williams^F^	4493–42-9	92	182.2629	0.02800	3.60	5.92	−0.46	1.16	0.500	
Fatty acid ester	**hexyl-2-methyl-butanoate**	*Malus domestica* cv. Discovery^A^	10,032–15-2	95	186.2947	0.15800	10.20	7.28	0.04	0.89	0.345	
Aromatic ester	**methyl salicylate**	*Malus domestica* cv. Discovery^A^	119–36-8	99	152.1494	0.03430	−3.60	3.92	−0.75	0.51	0.273	
Acetate ester	**(*E*,*E*)−8,10-dodecadien-1-yl acetate**	*C. pomonella*^E^	53,880–51-6	84	224.3437	0.00100	3.80	5.43	−0.33	1.00	0.345	
Acetate ester	**(*Z*,*Z*)−3,13-octadecadien-1-yl acetate**	Novel: main pheromone of *Synanthedon myopaeformis*	53,120–27-7	96	308.5053	Unknown	6.20	6.54	−0.20	1.11	0.345	
Sesquiterpene	**(*E*)-β-farnesene**	*Malus domestica* cv. Discovery^A^	18,794–84-8	90	204.3563	0.01000	2.80	4.47	−0.26	0.56	0.068	
Sesquiterpene	**(*E*,*E*)-α-farnesene**	*Malus domestica* cv. Discovery^A^	502–61-4	90	204.3563	0.01000	3.60	4.52	−0.10	0.43	**0.043**	*
Sesquiterpene	**(*E*)-β-caryophyllene**	*Malus domestica* cv. Discovery^A^	87–44-5	98.5	204.3563	0.01300	0.80	4.81	−0.49	0.64	0.893	
Cyanide	**2-phenylacetonitrile**	Novel	140–29-4	99	204.3563	0.01300	6.40	8.26	−0.46	1.15	0.686	
Acyclic monoterpenoid	**(*E*)−4,8-dimethyl-1,3,7-nonatriene**	*Malus domestica* cv. Discovery^A^	26,049–69-4	93	254.27	0.00032	2.80	4.55	−0.35	0.72	0.500	
Solvent	**hexane**	-	110–54-3	97	86.1776	151.00000	−1.60	3.31	−0.52	0.50	-	

Based on the database of odorant responses (http://neuro.uni-konstanz.de/DoOR/content/DoOR.php; Münch and Galizia 2011; Galizia et al. 2010), the panel also included 2-heptanone (CAS 110–43-0) and 3-octanol (CAS: 589–98-0) as positive controls to validate recordings from ab3 sensilla by testing activation of *D. melanogaster* ab3B. To discriminate ab3 from ab2 sensilla, the ab2A activator ethyl acetate (CAS: 141–78-6) was included as a negative control. To test absence in the ab3A neuron of the wild-type expression of the *D. melanogaster* OR22a/b-subunits, ethyl hexanoate (CAS 123–66-0) was included as an additional negative control.

To screen the panel, all odorants were diluted in hexane (Sigma Aldrich, St. Louis, MO-USA) at 1.0 μg/μL. Stimuli were prepared by applying 10.0 μL of each dilution on grade 1—20 mm circles filter paper (GE Healthcare Life Science, Little Chalfont, United Kingdom), previously inserted into glass Pasteur pipettes (VWR, Milan, Italy), for a total amount of 10.0 μg of compound per stimulus. To minimize possible effects from the solvent, pipettes were left at least 10 min after preparation under the fume hood for solvent evaporation. Puffing provided an additional 2.5 mL of air through the pipette for 0.5 s by inserting the pipette within a side hole of the glass tube, directing the humidified air flow to the antennae. To characterize the intensity of the response, spike frequency was calculated as in Lebreton et al. (2017) by subtracting ab3A spikes counted for 0.5 s before the stimulus from the number of spikes counted for 0.5 s after the stimulus to calculate spike frequency in terms of ∆spikes/0.5 s. Responses to compounds of the panel were compared for five replicates, using a single insect as a replicate. Before validating significant differences in spike counting, tests of normality with the IBM SPSS Statistics software 29.0 (https://www.ibm.com/) unveiled that for some ligands, data were not normally distributed (Kolmogorov-Smirnova/Shapiro–Wilk test p < 0.05, Supplementary Data File 3). Using the same software, spike frequencies of each compound were compared with respective values from the solvent (hexane) by the non-parametric Wilcoxon Signed Rank Test (p < 0.05). For box-plot analysis, ∆spikes/0.5 s of each recording was normalized to the averaged ab3A firing rate for the specific insect replicate, as done in our previous studies (Cattaneo et al. 2023). In brief, for every insect replicate, raw spiking (∆spikes/0.5 s) from all the tested ligands and the solvent were averaged to use such value as a representative for the single insect for normalization of the raw effects from each ligand (Supplementary Data File 3).

### Gas Chromatography Coupled with Single Sensillum Recordings (GC-SSR)

GC-SSR was performed with the same GC equipment in our labs that interfaced with the SSR rig we used in our previous investigation (Cattaneo et al. 2023). In brief, samples were injected on a 7890 GC-system (Agilent Technologies Inc., Santa Clara, CA, USA) provided with a 30 m × 0.32 mm fused silica capillary column (Agilent Technologies Inc.), coated with HP-5, df = 0.25 µm, programmed from 30 °C (hold 3 min) at 8 °C/min to 250 °C (hold 5 min) (software: GC-SSR-1—Agilent.OpenLab, Agilent Technologies). The outlet split from the GC column was a 1:1 ratio between the flame ionization detector (FID) and the mounted antenna, according to instrument settings. A humidified flow of 3.5–4.0 L/min charcoal-filtered air was directed into a 90-degree-angled glass tube with a hole on the angle where part of the column exiting from the transfer line was accessed. Glass-tubing was adjusted to a length of 17 cm, and ab3 sensilla was tested following the same optimization to 1.0 nanogram of active compound that we have adopted in Cattaneo et al (2023). The recording window was set to 35 min upon preliminary observation of retention times for the injected compounds. By GC-SSR we tested 1.0 ng and 10.0 ng aliquots of nonanal and (*Z*)−6-undecenal (*N* = 3) that we have chosen from our SSR-screening based on their effects (Table 1). Compounds were diluted in hexane between 0.001 and 0.010 μg/μL depending on the experiment condition, injecting 2.0 μL dilutions into the gas-chromatograph. Parallel experiments tested volatile collections from apple headspace (Hoplomalus, N = 7; Malus, *N* = 5) already available in our labs. To test headspace collections by GC-SSR, aliquots of 4.0 μL were injected into the gas chromatograph.

The headspace components that invoked responses were validated by recording in the GC-SSR using a DB-wax column. In brief, 2.0 μL of headspace was injected in the GC (7890) with an injector temperature of 225 °C in splitless mode. The GC was fitted with a silica capillary column coated with DB-wax column (Agilent Technologies Inc., df = 0.25 μm) and temperature programmed from 30 °C (hold time 3 min) until 225 °C (hold time 8 min) at 8 °C/min.

Hydrogen gas was used as a mobile phase at 2.7 mL/min. The GC effluent was carried onto the antennal preparation through a Gerstel ODP-2 transfer line connected to a glass tubing as described above. Retention times associated with neuronal activation were collected from the chromatograms exported from Chemstation B.03.02 (Agilent Technologies, Santa Clara, CA-USA) or from Autospike. To estimate neuronal activation, spikes were counted within 5 s from the emission of their respective GC peaks or the beginning of the ab3A effect, depending on the case. These counted spike numbers were subtracted from spikes five seconds antecedent to the effect and divided by 5 to calculate ∆spikes/second. Statistical analysis was performed as described above, including tests of normality (Supplementary Data File 3).

### GC–MS Analysis of Active Headspace

The volatile samples were injected on a GC–MS (Agilent technologies, 7890B GC coupled with 5977 MSD) equipped with a DB-WAX capillary column (60 m × 250 μm × 0.25 μm). A volume of 2.0 μL samples were injected into the injection port, set at 225 °C in splitless mode. The carrier gas was helium, and the total column flow was 1.883 mL/min. The temperature program of the oven started at 30 °C, which was held for 3 min and heated up at the rate of 8 °C/min to 225 °C, holding for 10 min at the final temperature. To perform GC–MS, we used the mass spectrometer in electron ionization mode at 70 eV, and the detector scanned in the 29–400 mass-to-charge range.

The volatile samples were also injected on a GC–MS (Agilent technologies, 6890 GC coupled with 5975 MSD) equipped with an HP-5 capillary column (column: 60 m × 250 μm × 0.25 μm). The column, inlet, and mass spectrometer settings were the same as those described above. The oven temperature program started at 50 °C, it was held for 2 min, and increased to 250 °C at the rate of 8 °C/min, holding the final temperature for 10 min. The same method was used to estimate ligand purities for compounds reported on Table 1 and others used in this study, including lactones tested on CpomORs expressed in *Xenopus* oocytes (next section, Supplementary Data File 4).

GC–MS data were analyzed using Agilent Mass Hunter B.08.00. The area counts were calculated using manual integration. The volatile components were tentatively identified by comparing the experimental mass spectra to those found in MS Libraries (NIST11 and Wiley12) using the Nist MS Search v. 2.4 program. The spectrum-based identification was verified by calculating the Kováts retention indices (RI) of components and comparing those to Kováts retention indices found in the NIST WebBook (https://webbook.nist.gov/) or PubChem databases.

### Cloning and Heterologous Expression of *CpomOR22* in Oocytes from *Xenopus laevis*

To further investigate the responsiveness of *CpomOR22* to environmental volatiles, we expressed *CpomOR22* in conjunction with the obligate coreceptor, *C. pomonella* Orco (*CpomOrco*), in unfertilized, defolliculated *X. laevis* oocytes. The main objectives of the *Xenopus* experiments were to validate positive findings from the *Drosophila* empty neuron system and expand the odor space/set of odorants used to test OR22, beyond those used in the SSR and GC-SSR experiments.

Using the two-electrode voltage clamp (TEVC) technique, we recorded responses of the odorant receptor complex, observed by the change in inward current, to 16 different odorant blends. These blends together contain 138 compounds, including 15 that were screened in *Drosophila*, grouped by chemical classes based on structure (Supplementary Table 2). The reason we have chosen this method was to make use of a parallel experimental setup based on a different heterologous system, in order to test a broader range of ligands within blends. Among these ligands, we kept nonanal as a reference, based on our observed SSR responses from this ligand being one the most active, which was also validated in the headspace from apple. The individual compounds, each at a concentration of 10^–4^ M in the blends, were applied to the oocyte with buffer perfusion.

*CpomOrco* and *CpomOR22* templates were synthesized by Twist Bioscience (South San Francisco, CA, USA) and delivered in pENTR vector, and then subcloned into the *X. laevis* compatible destination vector pSP64t (Gateway™ LR Clonase™ II Enzyme Mix, Invitrogen Corp., Carlsbad, CA, USA). Plasmids were purified using GeneJET Plasmid Miniprep Kit (ThermoFisher Scientific, Waltham, MA, USA) and verified using bidirectional Sanger sequencing (Psomagen, Rockville, MD, USA). 5,000 ng of each plasmid was linearized using *XbaI* (FastDigest *XbaI*, ThermoFisher Scientific, Waltham, MA, USA). The linearized plasmids were purified (GeneJET Gel Extraction Kit, ThermoFisher Scientific) and checked for concentration using the Nanodrop (NanoDrop™ One UV–Vis Spectrophotometer, Witec AG, Sursee, Switzerland). cRNA was synthesized from the linearized plasmids (mMESSAGE mMACHINE™ SP6 Transcription Kit, Themo Fisher Scientific, Waltham, MA, USA) and incubated at 37 °C for 12 h. 1.0 uL of RNase inhibitor was added to each reaction (RNaseOUT™ Recombinant Ribonuclease Inhibitor, ThermoFisher Scientific, Waltham, MA, USA). Synthesis was verified using gel electrophoresis, and concentration was checked using the Nanodrop (Thermo Fisher Scientific).

Stage V-VII *X. laevis* defolliculated oocytes were ordered from Xenopus1 (Dexter, MI, USA) and incubated in ND96 incubation media (96 mM NaCl, 2 mM KCl, 5 mM HEPES, 1.8 mM CaCl_2_, 1 mM MgCl_2_, pH 7.6) augmented with 5% horse serum (ThermoFisher Scientific, Waltham, MA, USA), 50 μg/mL tetracycline, 100 μg/mL streptomycin, 100 μg/mL penicillin, and 550 μg/mL sodium pyruvate. Oocytes were injected with 30 nL of RNA (30 ng of each cRNA) using the Nanoliter 2010 injector (World Precision Instruments, Inc., Sarasota, FL, USA). The resting membrane potential and odorant-induced changes of oocytes expressing odorant receptor cRNAs were recorded 72 h post-injection using the TEVC technique. The OC-725C oocyte clamp (Warner Instruments, LLC, Hamden, CT, USA) held a − 80 mV holding potential. The effect of the blends (Supplementary Data File 4) on odorant receptor complexes was determined by perfusing 10^−4^ M concentration blends across individual oocytes. The membrane current was permitted to return to baseline between blend introductions. Data was recorded with the Digidata 1550 B digitizer and pCLAMP10 software (Molecular Devices, Sunnyvale, CA, USA). Raw data was collected and normalized according to the blend or compound producing the greatest response (Supplementary Data File 4). Following initial screenings, unitary compounds comprising activating blends were perfused to establish tuning curves. Concentration responses for γ-undecalactone and undecanal were determined by challenging oocytes with half-log concentrations between 10^−7^ M and 10^−4^ M for 10 s. The current was allowed to return to baseline between compound administrations. Blends and unitary compounds were perfused on 8–10 oocytes per trial. GraphPad Prism 8 (GraphPad Software, Inc., La Jolla, CA, USA) was utilized for data analyses.

### Phylogenetic Analysis of *CpomOR22*

For an evolutionary assessment of the *CpomOR22* protein, a phylogenetic analysis was performed on *CpomOR22* in the context of OR receptors from *C. pomonella* and repertoires from other lepidopteran insect species. The phylogeny was built from OR sets from *C. pomonella* (Walker et al. 2016, 2023) *B. mori* (Wanner et al. 2007; Briscoe et al. 2013), *E. postvittana* (Corcoran et al 2015)*, Helicoverpa armigera* (Zhang et al. 2015)*, Lampronia capitella* (Yuvaraj et al. 2018)*, M. sexta* (Koenig et al. 2015) and *Spodoptera littoralis* (Walker et al. 2019). Amino acid sequences for each gene family were aligned using MAFFT online version 7.220 (https://mafft.cbrc.jp/alignment/server/, accessed on 30 January 2025) through the FFT-NS-I iterative refinement method, with JTT200 scoring matrix, “leave gappy regions” set, and other default parameters. Aligned sequences were used to build the unrooted phylogenetic tree using PhyML 3.0 (http://www.atgc-montpellier.fr/phyml/, accessed on 30 January 2025) (Guindon et al. 2010) using the BioNJ algorithm and maximum likelihood tree with Smart Model Selection (SMS) method (Lefort et al. 2017) with selection criterion set to the Bayesian Information Criterion. This software tool integrated into the PhyML web server automatically selects the best substitution model. For the OR phylogeny, the *JTT* + *R* + *F* model was selected. PhyML uses both NNI (nearest neighbor interchanges) and SPR (subtree pruning and regrafting) methods to rearrange and optimize the tree structure. Clade support for maximum likelihood analysis was assessed using the Shimodiara–Hasegawa approximate likelihood ratio test (SH-aLRT) (Anisimova and Gascuel 2006). The nodes with support values SH-aLRT > 0.9 were considered well supported, nodes with values ranging from 0.8 to 0.9 were considered weakly supported, and node values < 0.8 were considered unsupported (Guindon et al. 2010). A consensus Newick format tree was visualized and processed in MEGA-XI software (version 11.0.10) (Tamura et al. 2021) and the final tree output was edited with Adobe Illustrator (version 27.9).

## Results

### Expression Analysis of *CpomOR22* in *C. pomonella*

**Fig. 1:**
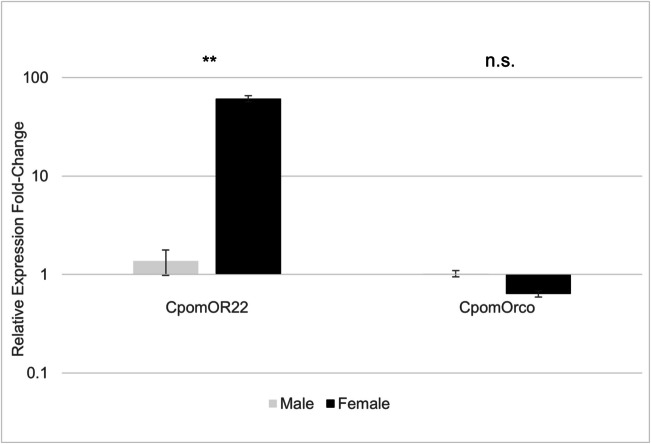
qRT-PCR expression fold change of *CpomOR22* and *CpomOrco* in female and male antennae. Relative gene expression fold-change values are shown on a log10 scale, normalized to reference genes (*ActR2* and *HSP40*), with average values of three biological replicates for each gene and tissue type shown. For each gene and biological sample, ∆(CT) values were calculated against the average CT of all male samples. Hence, values greater than one indicate higher expression in females, and values less than one indicate higher expression in males. Statistical assessments of differences in values between log2 transformed male and female values were conducted with Student's t-test (a two-tailed distribution with two-sample equal variance); "**" indicates a p-value less than 0.002. “n.s.” indicates “not significant” with a p-value greater than 0.05. Error bars are standard error values. Raw and normalized data are shown in Supplementary Data File 1

Our qRT-PCR study confirmed the previously non-replicated transcriptomic studies that indicated female bias for *OR22*, named *OR15* in Bengtsson et al. (2012), and afterward changed to its current nomenclature in Walker et al. (2016). *CpomOR22* was expressed in female antennae at a 44.28-fold higher level than male antennae (*p* = 0.0019; Fig. 1, Supplementary Data File 1). As a point of comparison, the odorant receptor coreceptor (*CpomOrco*) was observed to be expressed in female antennae at a 1.61-fold lower level than in male antennae without a statistically significant difference (*p* = 0.078).

### Functional Characterization of *CpomOR22* in Transgenic *Drosophila*

**Fig. 2:**
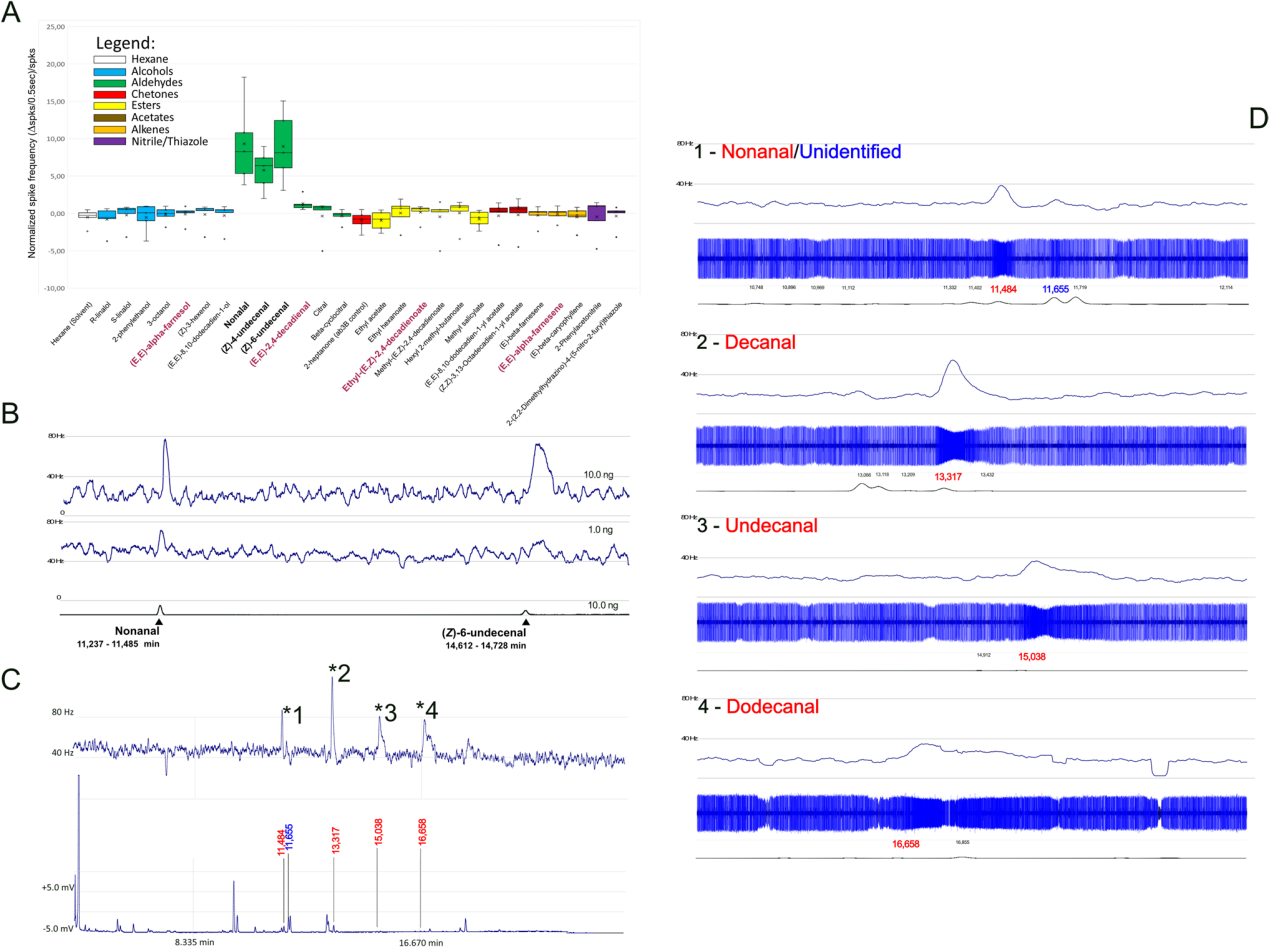
SSR and GC-SSR effects from ab3A neurons of transgenic *Drosophila* expressing *CpomOR22*. **A.** Boxplot of normalized ab3A spiking from transgenic *D. melanogaster* expressing *CpomOR22*, tested with the compound library reported in Table 1. Colors depict ligands belonging to different compound classes (see legend and Table 1). Bold-font compounds were significantly active on *CpomOR22*, with slight activators indicated with Magenta. Raw and normalized data from spike counting are shown in Supplementary Data File 3. **B.** GC-SSR experiment conducted by injecting GC-system provided with capillary column coated with HP-5, using 10.0 ng and 1.0 ng of nonanal [CAS: 124–19-6] and (*Z*)−6-undecenal [CAS: 60,671–73-0]. Note: frequency plots (above) summarize effects associated with 10 ng and 1.0 ng, while the gas-chromatogram shown below indicates peaks related to only 10.0 ng. **C.** GC-SSR experiment conducted by injecting the apple headspace of Hoplomalus 583 (Table 2, replicate 7); above, frequency plot associated with active components: 1 – nonanal/unidentified, 2 – decanal, 3 – undecanal, 4 – dodecanal; below, retention times (red/blue, min) identified in the GC-track. **D.** Magnification of the frequency plot (above), spiking effect (middle), and GC-FID (below) of each Section (1 to 4) selected from C. Retention times (min) are indicated as in C, and if not active, are shown in black

Testing a panel of possible ligands based on previous knowledge of compounds active on ORs/OSNs of *C. pomonella* (Table 1), SSR screening unveiled the aldehydes nonanal, (*Z*)−4-undecenal and (*Z*)−6-undecenal as the most active (Fig. 2A). In parallel, the Wilcoxon Signed Rank Test revealed slight activating differences from the effect of the solvent when testing other compounds, including ethyl-(*E*,*Z*)−2,4-decadienoate and the corresponding *E*,*E*-isomeric aldehyde, (*E*,*E*)−2,4-decadienal (0,15 ± 0,51 and 1,31 ± 0,42 normalized spikes/0.5 s respectively). Conversely, the sesquiterpenoid alcohol (*E*,*E*)-α-farnesol (−0.1 ± 0.53 normalized spikes/0.5 s) and its corresponding sesquiterpene (*E*,*E*)-α-farnesene (−0.1 ± 0.43 normalized spikes/0.5 s), when compared with hexane (−0.52 ± 0.5 normalized spikes/0.5 s), yielded an increment in the spiking effect which, despite being low, was significant (Wilcoxon Signed Rank Test, *p* = 0,043, α = 0.05) (Table 1).

To test the validity of *CpomOR22* responses we selected the most active aldehydes, nonanal (9.30 ± 2.53 normalized spikes/0.5 s) and (Z)−6-undecenal (8,97 ± 2.15 normalized spikes/0.5 s), for GC-SSR injections with minimal dosages expected from the sensitivity of our optimized equipment ([1.0–10.0] ng). This choice was based on our previous findings (Cattaneo et al. 2023) suggesting GC-SSR injection with synthetic compounds as a good laboratory practice before undertaking experiments with headspace extracts. For both the saturated (nonanal) and the most active unsaturated [(*Z*)−6-undecenal] aldehydes, for both dosages tested, an increment of associated neuronal spike frequency was observed (Fig. 2B).

### *C. pomonella* OR22 Responds to γ-Undecalactone and Undecanal in the *Xenopus* Oocyte System

**Fig. 3:**
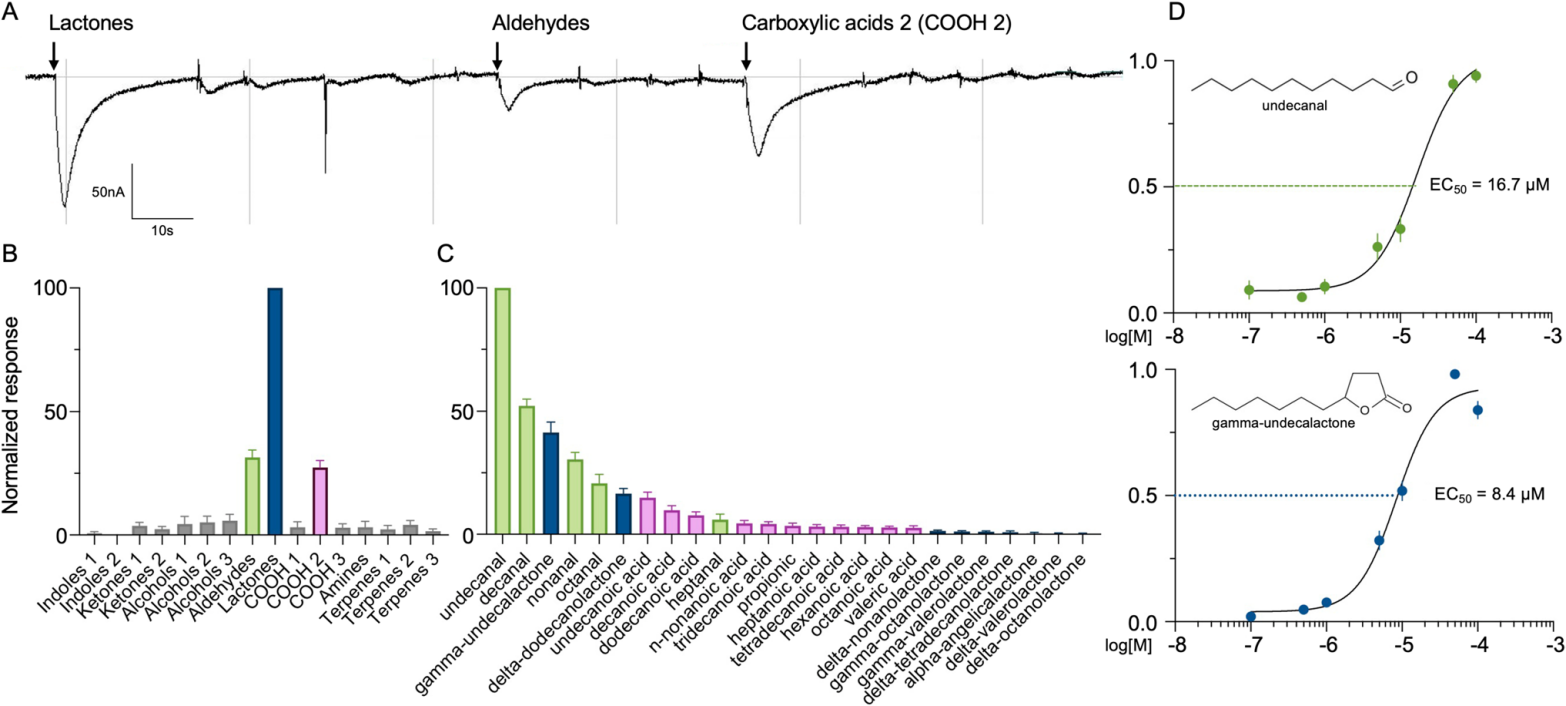
Functional characterization of *CpomOR22*/CpomOrco heterologously expressed in oocytes from *X. laevis*. Oocytes from *Xenopus* injected with *CpomOR22*/*CpomOrco* respond to environmentally relevant compounds. **A.** Trace from the TEVC software shows the changes in a single oocyte internal membrane potential. **B.** The averaged, normalized responses of 10 individual oocytes to exposure of the 16 compound blends. **C.** The averaged, normalized response of 10 individual oocytes to exposure of the individual components of the relevant compound blends. Note: the two most active lactones (gamma-undecalactone and delta-dodecalactone) and two other lactones, among the less active (delta-nonalactone and gamma-octalactone) were analyzed by GC–MS following standards indicated in methods confirming the absence of aldehydes contaminants and validating lactones purities ranging between 98 and 99%. **D.** The averaged, normalized responses of 10 individual oocytes to exposure of half log concentrations of undecanal (top) and γ-undecalactone (bottom) ranging from 10^–7^ to 10^–4^ M

Oocytes expressing the combination of *CpomOR22* & *CpomOrco* were stimulated with blends of compounds, organized by chemical class, to increase the number of compounds tested (Supplementary Table 2). Oocytes responded best to a blend of lactones, while also responding to blends of aldehydes and carboxylic acids (Fig. 3A, B). Subsequently, we tested each component from the three blends that produced the highest receptor complex activation. Individual compounds that elicited the highest activation were undecanal and decanal from the aldehyde blend, γ-undecalactone and δ-dodecalactone from the lactone blend, and undecanoic and decanoic acids from the carboxylic acid blend #2 (Fig. 3C). It is interesting to note that lactones produced a higher normalized response in blends than aldehydes, yet individual aldehydes, e.g., undecanal and decanal, elicited higher normalized responses than lactones (Fig. 3B, C). One possible explanation for this apparent contradiction is that lower efficacy aldehydes in the blend, such as heptanal and octanal, compete for the same ligand binding site in *CpomOR22* as undecanal and decanal, thus reducing the overall response magnitude in the context of the blend (Rospars et al. 2008; Singh et al. 2019). This blend effect may be less pronounced in the lactones. The similar carbon chain lengths of the most efficacious compounds from each blend suggest a conserved activation mechanism. Next, we examined *CpomOR22* dose responsiveness to undecanal and γ-undecalactone across five orders of magnitude and calculated the effective concentrations at the half-maximal response, EC_50_, which were 16.7 μM 8.4 μM, respectively (Fig. 3D).

### Identification of Active Ligands from Apple Headspace

**Fig. 4:**
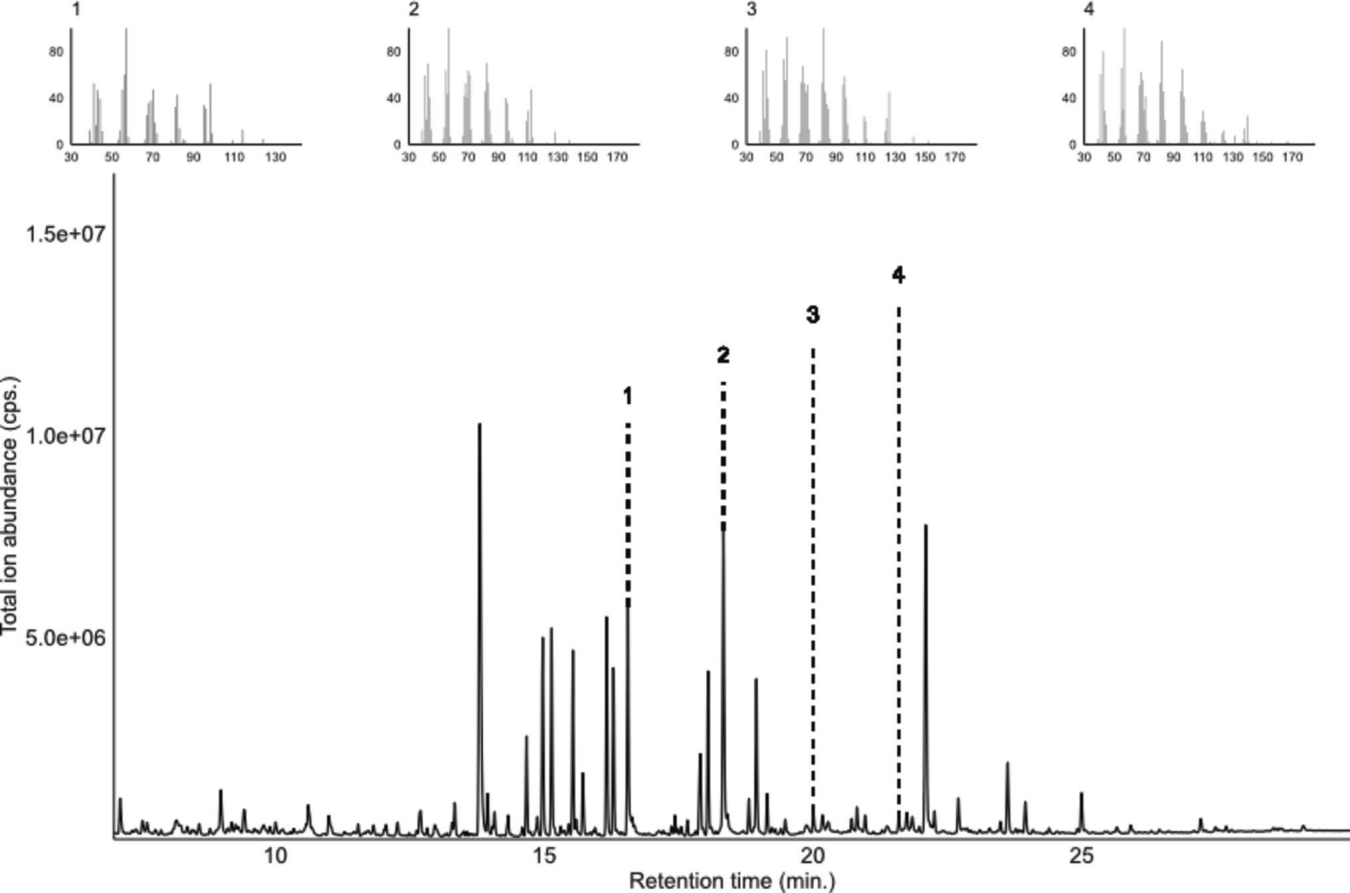
Total ion chromatogram of *H. testudinea* infested apple volatile collections separated on DB-WAX capillary column. X-axis: retention time (min); Y-axis: total ion abundance. The tentatively identified sensillum-active components are nonanal (1), decanal (2), undecanal (3), dodecanal (4)

The Hoplomalus and Malus volatile extracts were injected on both polar (DB-WAX) and non-polar (HP-5) capillary columns and in both cases, four components elicited consistent electrophysiological responses. The calculated Kováts retention indices of these components were 1393, 1498, 1606 and 1713 using the DB-WAX capillary column and 1105, 1206, 1307 and 1409 using HP-5 capillary column. We have analyzed the volatile extracts using GC–MS on both polar (DB-WAX) and non-polar (HP-5) capillary columns and identified active components based on their mass spectra and Kováts indices, which represented four saturated aldehydes: nonanal [DB-wax: RT: ~ 11.50 min; kováts ~ 1395; HP-5: RT: ~ 11.48 min; kováts ~ 1105], decanal [DB-wax: RT: ~ 13,15 min; kováts ~ 1499; HP-5: RT: ~ 13,32 min; kováts ~ 1207], undecanal [DB-wax: RT: ~ 14.78 min; kováts ~ 1608; HP-5: RT: ~ 15.04 min; kováts ~ 1308] and dodecanal [DB-wax: RT: ~ 16.30 min; kováts ~ 1716; HP-5: RT: ~ 16.66 min; kováts ~ 1410] (Fig. 2C, D, Fig. 4). In addition, one component that was only visible on the non-polar (HP-5) column with retention time proximal to 11.66 min (Kováts retention index 1114) was also active on *CpomOR22* (6,15 ± 1,21 spikes/sec), but it remained unidentified due to low peak abundance and coelution of multiple peaks (Fig. 2C, D; Table 2).

**Table 2:** GC-SSR spike counting for responses to Hoplomalus (Hopl) and Malus (Mal) headspace. Raw (Spikes/5.0 s), spike counting (spikes/sec), and parameters from GC-SSR runs (retention times, RT and Kováts index, Kov.) testing Hoplomalus and Malus headspace on DB-wax (Rep1-4) and HP-5 columns (Rep 5–12) are available in Supplementary Data File 3

Spike counting (Spikes/sec)															
Headsp.	Hopl 583			Mal 590	Hopl 566	Hopl 560	Hopl583	Hopl500	Mal 561		Mal 559	Mal 603	Wilcoxon Signed Rank Test		
*GC–MS*	**R1**	**R2**	**R3**	**R4**	**R5**	**R6**	**R7**	**R8**	**R9**	**R10**	**R11**	**R12**	**Avrg**	**Std.Er**	***p-val***
hexane	3.0	6.0	3.0	13.0	1.0	0.2	0.2	−0.2	2.8	−1.6	−0.8	2.6	2.43	1.14	-
*nonanal*	16.8	2.8	19.0	27.4	36.0	8.4	17.6	3.2	12.8	13	3.6	10.8	14.28	2.89	**0.003**
*unknown*	-	-	-	-	12.8	5.2	8.0	2.6	8.2	5.8	3.8	2.8	6.15	1.21	**0.012**
*decanal*	19.2	22.8	22.0	42.4	53.8	16.8	51	−2.6	14.6	24	17.2	29.2	25.87	4.66	**0.003**
*undecanal*	33.0	9.8	8.8	21.0	43.6	11.0	22.6	−1.2	9.6	10.6	8.0	16.4	16.10	3.55	**0.003**
*dodecanal*	28.6	11.2	16.6	25.4	46.0	9.4	27.4	3.0	7.6	5.2	4.4	9.4	16.18	3.77	**0.002**
Column	DB-wax				HP-5										

Among the saturated aldehydes, decanal was the most active (25.87 ± 4.66 spikes/sec), followed by dodecanal and undecanal, with similar values (16.18 ± 3.77 spikes/sec and 16.10 ± 3.55 spikes/sec respectively) and nonanal (14.28 ± 2.89 spikes/sec). Performing a Wilcoxon Signed Rank Test comparison with the effect elicited by the solvent demonstrated all these ligands were significantly active when they reached *D. melanogaster* ab3A neurons expressing *CpomOR22*, with the lowest *p*-value for dodecanal (*p* = 0.002).

## Discussion

While much focus has deservedly been placed on studying the function of male-specific or male-biased candidate PRs of Lepidoptera, more attention should be given to the female-specific and female-biased ORs. In this study, we isolated, heterologously expressed, and functionally characterized *CpomOR22*, previously reported as a candidate pheromone receptor and having a clear female-antenna expression bias (Walker et al. 2016). Functional characterization performed using SSR and GC-SSR on transgenic *D. melanogaster* demonstrated binding of this OR to a few ligands, including aldehydes emitted from apple headspace. An *in vitro* approach using oocytes from *Xenopus* has been brought in to confirm response to aldehydes and to expand the chemical space for testing, confirming binding to these ligands and adding evidence of responses to lactone structural analogues.

We initially hypothesized that *CpomOR22* could be a pheromone receptor, based on its clustering within the canonical lepidopteran PR clade (Zhang and Löfstedt 2015). We did not observe responses of this OR to codling moth pheromone compounds. However, activation of OR22 by long chain fatty acid derivatives (in this case aldehydes and lactones) is generally consistent with the ligand tuning of ORs within the canonical PR clade (Zhang and Löfstedt 2015; de Fouchier et al. 2017; Yuvaraj et al. 2018). This stands in contrast to lepidopteran non-PR clade ORs responsive to plant compounds that display within-clade conserved responses to other types of odorants such as aromatics and terpenes (de Fouchier et al. 2017).

Within the PR-clade CpomOR22 is part of a sub-family that includes *E. postvittana* OR22 (EposOR22), *M. sexta* OR15 and OR51 (MsexOR15/MsexOR51), and *B. mori* OR6 (BmorOR6); (Supplementary Fig. 1). Among this sub-family, CpomOR22, EposOR22 (Corcoran et al. 2015) and MsexOR15 (Koenig et al. 2015; Tom et al. 2022) display female-biased or specific expression in moth antennae, while BmorOR6 (Wanner et al. 2007) and MsexOR51 (Koenig et al. 2015; Tom et al. 2022) display male-biased or specific expression. This heterogeneity raises questions about the evolution of PRs among ORs that detect plant volatiles or *vice versa*.

CpomOR22 is female-biased and detects plant volatiles; to our knowledge, none of the other ORs in this sub-family have been functionally characterized, though efforts to identify ligands for EposOR22 were not successful (Yuvaraj et al. 2022). Three odorant receptors from the non-ditrysian moth, *L. capitella,* LcapOR3/LcapOR5/LcapOR7, clustered basally to the sub-family with CpomOR22. One of these, LcapOR7 has been characterized as a pheromone receptor (Yuvaraj et al. 2018), indicative of the ancient origins of emergence of PRs within this clade. Conversely, in a more basal non-ditrysian moth, *Eriocrania semipurpurella*, ORs within the canonical PR clade were not identified. However, a separate subfamily of ORs was identified, in which EsemOR3 and EsemOR5 respond to pheromones and plant compounds (Yuvaraj et al. 2017), suggesting that PRs in this clade may have evolved from ORs responsive to plant compounds, while EsemOR1, which is basal to the canonical PR clade, is responsive to plant compounds (Yuvaraj et al. 2017). From this, it may be hypothesized that CpomOR22 and other female-specific or biased ORs within the canonical PR clade may have retained their ancestral function, i.e. responsiveness to plant compounds. Our study demonstrates binding of the candidate pheromone receptor CpomOR22 to both unsaturated and saturated aldehydes and it adds to the knowledge of volatile compounds known for being emitted by hosts and active on the olfactory chemoreceptor systems of *C. pomonella*, such as pear ester ethyl-(*E*,*Z*)−2,4-decadienoate (Bengtsson et al. 2014; Cattaneo et al. 2017) (Fig. 2A).

While we recognize the importance to verify the impact on live-insects of ligands, such as apple-emitted aldehydes, that we have found active on CpomOR22, behavioral experiments are beyond the scope of this project at the present time. Moreover, previous chemical ecology research on *C. pomonella* has already investigated some of these aspects by demonstrating the role of aldehydes on live moths. Indeed, aldehydes like nonanal and decanal, have been part of comprehensive electrophysiological and behavioral investigations on *C. pomonella*, previously reported to generate maximal electroantennal responses in both sexes of this insect (Casado et al. 2006). Furthermore, nonanal was shown to increase oviposition in behavioral bioassays comparing synthetic plant volatiles to green apples (Witzgall et al. 2005). Other studies, however, using olfactometers with two selection Y-tube assays, identified nonanal among the repellent odors, the repellency of which was overcome when this compound was part of a more complex mixture of apple volatiles (Vallat and Dorn 2005). Conversely, the addition of aldehydes to pear ester did not increase the number of codling moth trap catches, for neither males or females, in walnut and apple orchards (Light and Knight 2005). Collectively, these studies highlight the complexities of insect chemical ecology as it relates to single compounds versus blends, and disparate roles in mediating different behaviors. Apart from their effects on adult codling moths, aldehydes are also among the minor components of the bouquet of larval aggregation pheromones (Jumean et al. 2005a). Beyond the codling moth, aldehydes are also part of a wide range of host semiochemicals that stimulate attraction to hosts and oviposition for gravid females of various insects from different taxa (Gonzalez 2007).

We recognize that in SSR-experiments, responses to pear ester are weaker than the responses to aldehydes for CpomOR22 (Fig. 2A). However, it deserves to be noted that the effects enhanced by this ligand are significantly different than spiking from the solvent (0,15 ± 0,51 normalized spikes/0.5 s; *p* = 0,043, Table 1). Interestingly, our SSR analysis unveiled the sesquiterpene (*E*,*E*)-α-farnesene and its analogue alcohol, (*E*,*E*)-α-farnesol as slight activators (−0.10 ± 0.43 and −0.10 ± 0.53 normalized spikes/0.5 s, *p* = 0.043; Supplementary Data File 3). (*E*,*E*)-α-farnesene is the most abundant compound in apple headspace and it is among the most potent elicitors of antennal and behavioral responses of codling moth females (Wearing and Hutchins 1973; Light et al. 2001; Ansebo et al. 2004; Coracini et al. 2003). Conversely, (*E*,*E*)-α-farnesol, which has been identified only in trace quantities from Granny Smith apples (Matich et al. 1996), has been reported to enhance the upwind flight of codling moth males (Ansebo et al. 2005; Coracini et al. 2003), consistent with findings that specific neuronal receptors responding to this ligand have been found only on male antennae (Ansebo et al. 2005). Collectively, these studies, together with our findings on CpomOR22, and evidence of lack of expression of this sensor in males (Walker et al. 2016), suggest that other antennal chemoreceptors from male antennae of *C. pomonella* may be involved in sensing (*E*,*E*)-α-farnesol, while our evidence of OR22-binding of (*E*,*E*)-α-farnesene, despite shown as a slight activation, seems to be in accordance with the reported evidence of its behavioral responses for females, even though other female-biased ORs may exist showing a more robust response to this compound.

Among the unsaturated aldehydes, we observed significant effects on CpomOR22 from (*E*,*E*)−2,4-decadienal (*p* = 0.043, Supplementary Data File 3), despite not being outstanding (1,31 ± 0,42 normalized spikes/0.5 s, *p* = 0,043; Table 1). Together with pear ester, this compound was reported among the ligands enhancing the highest electrophysiological responses when tested on female antennae, while males were repelled when (*E*,*E*)−2,4-decadienal was added in blends with other active compounds (Ansebo et al. 2004). The same study also revealed that (*Z*)−3-hexenol and (R/S)-linalool generated strong olfactory responses in female antennae. However, when tested by SSR on *D. melanogaster* ab3A neurons expressing CpomOR22, neither of these compounds elicited a significant response (*p* > 0.05, Supplementary Data File 3). Most likely, other CpomORs are involved in binding these ligands, and further heterologous studies are needed to search for candidates aimed at decrypting female-related mechanisms to detect the main apple-emitted semiochemicals. However, despite that averages of normalized responses to (*E*,*E*)−2,4-decadienal and pear ester are negligible when compared to the effects from the other aldehydes that we have tested, our reported CpomOR22 activation by these ligands is interesting because of their common effects on both males and females (Ansebo et al. 2004, 2005; Coracini et al. 2003). Indeed, while pear ester is attractive for both sexes of the codling moth, and has been used for decades primarily for main monitoring programs (Light et al. 2001), (*E*,*E*)−2,4-decadienal is electroantennographically active on females and behaviorally repulsive on males, despite that behavioral effects on females have yet to be tested (Ansebo et al. 2004). In addition, both ligands are renowned semiochemicals capable of interfering with the chemosensory systems of other insects, belonging or not to the order of Lepidoptera (Anfora et al. 2014; Wenda-Piesik et al. 2018). Evidence of CpomOR22 activation by ethyl-(*E*,*Z*)−2,4-decadienoate and (*E*,*E*)−2,4-decadienal may add to future functional characterization efforts for orthologues of this receptor from other species.

As mentioned, unsaturated aldehydes like (*Z*)−4-undecenal showed a more evident effect (Fig. 2 A, B). (*Z*)−4-undecenal is renowned among the aldehydes emitted from the autoxidation of *Drosophila*'s cuticular hydrocarbons, and it has been part of our previous pharmacological investigations on the OR69a-subunits of *D. melanogaster* (Lebreton et al. 2017) and *Drosophila suzukii* (Cattaneo et al. 2023). While the activation of CpomOR22 as a candidate pheromone receptor with a female bias (Walker et al. 2016), by aldehydes like (*Z*)−4-undecenal is intriguing, no ecological roles for (*Z*)−4-undecenal are yet known in association with codling moth. CpomOR22 activation by this ligand may result from a mere pharmacological effect due to its structural similarity with other aldehydes. Indeed, aldehydes are volatile compounds emitted by diverse sources, including plants, animals, microbes, and raw meat (Cui et al. 2023). Our experimental records demonstrated CpomOR22 versatility in response to various saturated and unsaturated aldehydes, including undecanal (Fig. 2D). Although hypothetically, unsaturated aldehydes from also nonanal, decanal, and dodecanal may represent possible ligands for CpomOR22, and future projects may attempt further deorphanization assays to resolve this. For instance, the unsaturated (E)−2-nonenal has been found to elicit similar behavioral responses as nonanal in larvae of *C. pomonella* (Jumean et al. 2005a); 2-decenal has been found in the diethyl-ether apple frass extract from caterpillars of *C. pomonella*, which was found to be attractive for the parasitic wasp *Hyssopus pallidus* (Gandolfi et al. 2003); the codlemone aldehyde (*E*,*E*)−8–10-dodecadienal is part of the female odor bouquet, and it was reported to attract males to field traps (Greenway 1984; Witzgall et al. 1993). The evidence in this report of the response to aldehydes by CpomOR22 opens the door to future experiments validating binding of this sensor to the codlemone aldehyde. Despite being hypothetical, such binding would indicate the role of CpomOR22 in female aggregation, or in facilitating host finding. We previously examined this concept in the context of activation of another *C. pomonella* pheromone receptor by other odorants that are part of the bouquet emitted by codling moth females (Cattaneo et al. 2017).

In addition to our main findings (Fig. 2A, B), we conducted supplementary experiments testing effects from the same transgenic *Drosophila* with doses of (*Z*)−6-undecenal ranging from 1 ng to 150 μg. We observed CpomOR22-associated SSR spiking to (*Z*)−6-undecenal with a sensitivity of 2.903 ± 1.709 µg (EC50), Fmax of 0.8772 ± 0.1129, Hill coefficient of 0.8466 ± 0.3356 and a saturation at 50 µg dose (Supplementary Fig. 2, Supplementary Table 3). Interestingly, 6-undecenal has been found only among the main constituents from the essential oils of coriander (*Coriandrum sativum*), though it was not specified if it was present either in the *E* or the *Z* isomer. In the content of these essential oils, 6-undecenal is present together with saturated aldehydes ranging from nonanal to dodecanal (Parthasarathy et al. 2008; Rajeshwari and Andallu 2011). Although limited research has been conducted on this topic, and to the best of our knowledge there is no documented research on ecological interactions between *C. pomonella* and *Coriandrum sativum*, recent experiments testing essential oils of coriander demonstrated their repulsive effects on *Tricogrammatiddae cacoeciae* (Cointe 2020), which is a renowned egg parasitoid of *C. pomonella* (Mansour 2019). Other studies also reported that nonanal and decanal are required in a minimal blend to attract another pupal parasitoid of the codling moth, *Mastrus ridibundus* (Jumean et al. 2005b). Although behavioral studies are necessary to demonstrate whether the aldehydes that we have found active on CpomOR22 are attractants or repellents for codling moth females and the natural enemies of this insect, evidence of their emission by host apple fruits and their reported identification in a non-host such as coriander is compelling. If on one side we may hypothesize that the identified aldehyde kairomone active on CpomOR22 may direct codling moth females on apples or being somehow responsible for the insects’ aggregation, the same ligands may also modulate egg or pupal parasitization of *C. pomonella* being either repulsive or (possibly) attractive on the natural enemies of this insect. Indeed, in their experiments, Gandolfi et al. (2003) demonstrated that *H. pallidus* parasitoids are attracted to both the frass extracts from larvae fed with apple food and the same apples’ extracts, validating that both samples shared content of four main aldehydes that are commonly emitted from apple (2-heptenal, nonanal, decanal, 2,4-decadienal). Among these aldehydes, nonanal, decanal and 2,4-decadienal are active on CpomOR22 (Fig. 2). To test whether these aldehydes may be sensed by the parasitoids of *C. pomonella*, electrophysiological and behavioral trials are needed on these insects to investigate their chemosensory systems. This would unveil the possible, though speculative, roles of aldehyde ligands in more complex ecological interactions.

The reported ecological complexity behind the interaction of aldehydes with the codling moth may involve additional mechanisms for their detection. While future projects may unveil the existence of other chemosensors with an antennal male bias binding aldehyde ligands, in a recent investigation we have described the chemosensory receptors repertoire of the female abdomen tip of the codling moth (Walker et al. 2023). Conducting an RT-PCR analysis from RNA extracts of the ovipositor and pheromone glands, we have identified traces of CpomOR22, suggesting possible roles for this receptor in guiding oviposition behaviors or having possible roles in courtship (Roscoe et al. 2016), though to our knowledge, no male courtship pheromones have been identified for *C. pomonella*.

Aside from our findings on nonanal and decanal, to the best of our knowledge, ours is the first study reporting activation of the *C. pomonella* olfactory system by undecanal and dodecanal (Fig. 2D). Undecanal and dodecanal together with nonanal and decanal have been shown to be emitted by both infested and non-infested apples (Hern and Dorn 2002) (Fig. 2D, Table 2). Given that essential oils from non-hosts, which contain these two aldehydes among the others, are repellent to egg-parasitoids of the codling moth (Cointe 2020; Rajeshwari and Andallu 2011), evidence of the CpomOR22-sensing of undecanal and dodecanal suggests broader ecological importance for parasitoid interactions with *C. pomonella* that deserves further investigation. Furthermore, in the apple headspace that we have analyzed, we have identified another activating ligand represented by a retention time proximal to 11.60 min (Kováts Retention Index 1114) in the HP-5 column, which, by GC–MS analysis, we were not able to characterize (Fig. 2C, D, Table 2). When conducting GC-SSR experiments setting the gas-chromatograph with a DB-wax column, this ligand was not released (Table 2), possibly because of polar incompatibility, as discussed in our recent investigation performing similar trials with the HP-5 column (Cattaneo et al. 2023). On the other side, it might be as well that this compound was present as a contaminant rather within the HP-5 column or within the inlet of the GC. Independent of any possible scenario, since activation of CpomOR22 by this unknown ligand is compelling and may hide additional aspects of the *C. pomonella* molecular ecology, possible trials to attempt its characterization are warranted and may lead to a better understanding of the ecological importance of the broad tuning we have so far demonstrated for this *C. pomonella* subunit.

Using the *Xenopus laevis* heterologous expression system, we confirmed *CpomOR22* response to nonanal, decanal, and undecanal. Among other tested ligands, from lactones to carboxylic acids, we observed higher responses to γ-undecalactone. Like undecanal, γ-undecalactone is an 11-carbon compound that is found, together with other lactones, in fruit and fruit product odors (Pérez-Olivero et al. 2014; Lasekan and Hussein 2018; Hijaz et al. 2016) and it has previously been identified as a sensory receptor agonist (Tobita et al. 2021; Bezerra-Silva et al. 2016), as well as an arthropod behavioral regulator (Phelan et al. 1986; Weeks et al. 2020; Leyrer and Monroe 1973). Although it is also possible that the response of *CpomOR22* to γ-undecalactone may be related to the similarity of its structure with the carbon chain of the undecanal and the presence of a double bond to oxygen (Fig. 3) instead of any ecological relevance for codling moth.

Using SSR and GC-SSR recordings, this study deorphanized an odorant receptor that we have previously identified as part of the pheromone receptor clade of the codling moth by performing its heterologous expression *in vivo* using empty neurons of transgenic *Drosophila*. Combining oocytes from *Xenopus*, we demonstrated the binding of some among the same ligands *in vitro*, including nonanal, decanal and undecanal aldehydes, and we unveiled activation by γ-undecalactone. Comparing the two systems we observed that responses to the aforementioned aldehydes, were different, yet similar, resulting in decanal giving a stronger effect then undecanal and nonanal in GC-SSR (Supplementary Data File 3) and undecanal giving a stronger effect then decanal and nonanal in *Xenopus* oocytes (Supplementary Data File 4). However, such *in vitro* system dependent differences may be expected, having been previously observed when comparing different heterologous systems expressing ORs from other moths (Hou et al. 2020) or bark beetles (Yuvaraj et al. 2021). The subsequent identification of lactones as ligands for *CpomOR22* deserves further investigation: additional research may shed light on whether these lactones would merely represent structural analogues to the ecologically relevant aldehydes or if they are novel semiochemicals not previously identified among the extensive body of research done on codling moth chemical ecology. In case of the latter, identification of sources for their emission would become necessary. Although it must be emphasized that the lactones we have found active may merely represent structural analogues, given their common absence from apple volatile headspace (Bengtsson et al. 2001). The potential of structural chemistry/analogs in establishing novel ligands was reported in other species (Douglass et al. 1993; Bengtsson et al. 1990; Gonzalez et al. 2015). Furthermore, it is not uncommon for insect ORs binding analogues; for example, the insect odorant receptor co-receptor binds the synthetic VUAA-agonist (Jones et al. 2011) but its natural ligand(s) remains unknown. Other findings reported also Orco-blockage by amiloride antagonists (Röllecke et al. 2013) despite that these ligands represent more general ion-channel blockers for the chemoreceptors of different arthropods (Bobkov and Ache 2007) suggesting that all insect or, perhaps, even all arthropod chemosensory receptor channels (including ORs) can be characterized by a somewhat common pharmacology (Cattaneo et al. 2017). Given the evidence that the aldehyde and lactone from our studies share a structural backbone and both activate *CpomOR22*, a structural analysis investigation may help prediction for novel ligands in the phase of studying insect chemoreceptors, as it has recently been demonstrated for other ORs from *C. pomonella* (Gonzalez et al. 2015) and from another moth, *S. littoralis* (de Fouchier et al. 2017; Caballero-Vidal et al. 2021).

Despite our working hypothesis based on the phylogenetic analysis investigated *CpomOR22* as a candidate pheromone receptor (Walker et al. 2016), evidence from our study suggested this subunit as a typical female-biased expressed OR, apparently not binding to sex pheromones (Fig. 2A, Table 1), but rather responding to various apple volatile kairomones. The potential ecological relevance of these kairomones for the codling moth requires future investigations to validate their influence on the behavior of *C. pomonella* and their potential as semiochemicals for integrated push–pull strategies.

## Supplementary Information

Below is the link to the electronic supplementary material.

Supplementary Data File 1 (XLSX 29 KB)

Supplementary Data File 2 (DOCX 17.3 KB)

Supplementary Data File 3 (XLSX 33.6 KB)

Supplementary Data File 4 (XLSX 43.9 KB)

Supplementary Table 1 (DOCX 14 KB)

Supplementary Table 2 (XLSX 20.6 KB)

Supplementary Table 3 (XLSX 16.1 KB)

Supplementary Figure 1 (PDF 437 KB)

Supplementary Figure 2 (PDF 486 KB)

Supplementary File 10 (DOCX 36.2 KB)

## Data Availability

No datasets were generated or analysed during the current study.

## Abbreviations

OR: Odorant Receptor
OSN: Olfactory Sensory Neuron
PR: Pheromone Receptor
SSR: Single Sensillum Recording
GC-SSR: Gas-Chromatographic SSR
GC-MS: Gas-Chromatography coupled with Mass-Spectrometry
TEVC: Two Electron Voltage Clamp
FID: Flame Ionization Detector
