# Supplementary material for: Functional Characterization of a Female-Biased Chemoreceptor of the Codling Moth (Cydia pomonella) Responding to Aldehydes and Other Volatile Compounds
Source: J Chem Ecol. 2025 Feb 25;51(2):28. doi: 10.1007/s10886-025-01579-1 (PMC11861427; doi:10.1007/s10886-025-01579-1)
Supplement: Supplementary file 5 — Supplementary Table 1 (DOCX 14 KB) [file 10886_2025_1579_MOESM5_ESM.docx]

**Functional characterization of a female-biased chemoreceptor of the codling moth (Cydia pomonella) responding to aldehydes and other volatile compounds**

Alberto Maria Cattaneo^1*#^, Charles A. Kwadha^1,2#^, Heidi Pullmann-Lindsley^3^, Anna L. Erdei^1^, R. Jason Pitts^3^ and William B. Walker III^1,4*^

^1^Swedish University of Agricultural Sciences, Department of Plant Protection Biology, Chemical Ecology. Lomma - Campus Alnarp, SE-234 56 Sweden

^2^North Carolina State University, Department of Entomology and Plant Pathology, 1575 Varsity Drive, NC 27695-7616, USA

^3^Baylor University, Department of Biology, 101 Bagby Avenue, Waco, TX 76706, USA

^4^USDA-ARS, Temperate Tree Fruit and Vegetable Research Unit, 5230 Konnowac Pass Road Wapato, WA, 98951, USA

*Corresponding Authors: albertomaria.cattaneo@gmail.com , william.walker@usda.gov

#Authors contributed equally

| Primer Name | Primer Sequence | Primer Pair qPCR Efficiency |
| --- | --- | --- |
| CpomOR22-F | TGGGAGAAGATGTCAGTACCT | 1.86 |
| CpomOR22-R | AGGCCTTACTCCAGCTCTTA | 1.86 |
| CpomActR2-F | CGAAGTTCAAGATCCGCATAGA | 1.68 |
| CpomActR2-R | CCAGAAGTTGTCCCTGTTCTT | 1.68 |
| CpomHSP40-F | GGCAGTGACATCAGACATACA | 1.72 |
| CpomHSP40-R | TAGCTTCTCTCCGGTCATAGT | 1.72 |
| CpomOrco-F | GTGTTTCACTTCTGCATCTTCG | 1.97 |
| CpomOrco-R | CTCATAGCCTTCTGACACTGC | 1.97 |
