# Supplementary figures and images for: Functional Characterization of a Female-Biased Chemoreceptor of the Codling Moth (Cydia pomonella) Responding to Aldehydes and Other Volatile Compounds
Source: J Chem Ecol. 2025 Feb 25;51(2):28. doi: 10.1007/s10886-025-01579-1 (PMC11861427; doi:10.1007/s10886-025-01579-1)

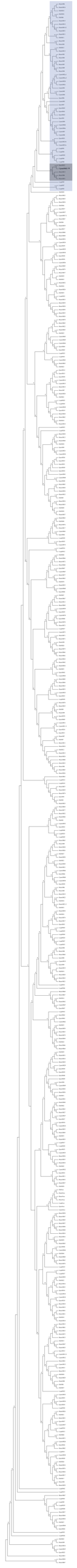

Supplement: Supplementary file 8 — Supplementary Figure 1 (PDF 437 KB) [file 10886_2025_1579_MOESM8_ESM.pdf]
