# Supplementary material for: Functional Characterization of a Female-Biased Chemoreceptor of the Codling Moth (Cydia pomonella) Responding to Aldehydes and Other Volatile Compounds
Source: J Chem Ecol. 2025 Feb 25;51(2):28. doi: 10.1007/s10886-025-01579-1 (PMC11861427; doi:10.1007/s10886-025-01579-1)

Functional characterization of a female-biased chemoreceptor of the codling moth (*Cydia pomonella*) responding to aldehydes and other volatile compounds

Alberto Maria Cattaneo[1*#], Charles A. Kwadha[1,2#], Heidi Pullmann-Lindsley[3], Anna L. Erdei[1], R. Jason Pitts[3] and William B. Walker III[1,4*]

[1]Swedish University of Agricultural Sciences, Department of Plant Protection Biology, Chemical Ecology. Lomma - Campus Alnarp, SE-234 56 Sweden
[2]North Carolina State University, Department of Entomology and Plant Pathology, 1575 Varsity Drive, NC 27695-7616, USA
[3]Baylor University, Department of Biology, 101 Bagby Avenue, Waco, TX 76706, USA
[4]USDA-ARS, Temperate Tree Fruit and Vegetable Research Unit, 5230 Konnowac Pass Road Wapato, WA, 98951, USA
*Corresponding Authors: albertomaria.cattaneo@gmail.com , william.walker@usda.gov

#Authors contributed equally

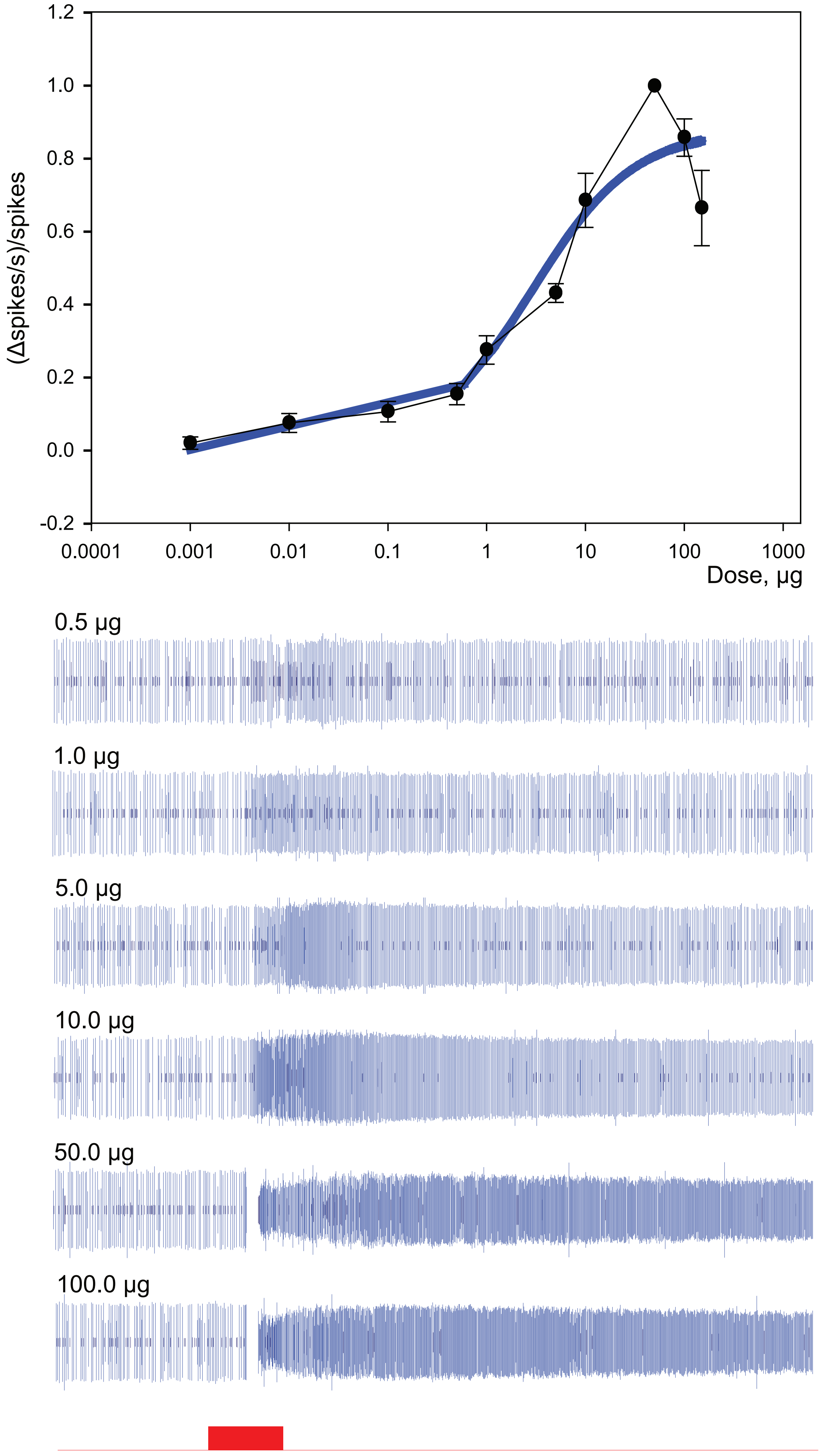

Supplement: Supplementary file 9 — Supplementary Figure 2 (PDF 486 KB) [file 10886_2025_1579_MOESM9_ESM.pdf]
