# Supplementary material for: Functional Characterization of a Female-Biased Chemoreceptor of the Codling Moth (Cydia pomonella) Responding to Aldehydes and Other Volatile Compounds
Source: J Chem Ecol. 2025 Feb 25;51(2):28. doi: 10.1007/s10886-025-01579-1 (PMC11861427; doi:10.1007/s10886-025-01579-1)
Supplement: Supplementary file 10 — (DOCX 36.2 KB) [file 10886_2025_1579_MOESM10_ESM.docx]

**Supporting information descriptions**

**Supplementary Data File 1**. qRT-PCR data file: raw CT values and relative quantity (RQ) values with target gene values normalized to the geometric mean of RQ values control genes; normalized gene expression values for each biological sample calibrated to average of gene expression values from male antennae samples; bar plot for normalized and calibrated relative gene expression fold-change is shown in Fig. 1 (XLSX).

**Supplementary Data File 2.** Open reading frame sequence information for *OR22*, including non-codon optimized nucleotide sequence (from the transcriptome of Walker et al. (2016)) used in *Xenopus* oocyte experiments, codon-optimized nucleotide sequence, optimized for *D. melanogaster* codon usage, used in *Drosophila* empty neuron SSR/GC-SSR experiments, and complete odorant receptor protein sequence (DOC).

**Supplementary Data File 3.** SSR-sheet: raw and normalized spike counting of compounds tested on CpomOR22 (Table 1), statistical analysis, including tests of normality, from normalized spike counting. GC-SSR sheet: raw and normalized spike counting of characterized retention time tested by GC-SSR on CpomOR22 (Table 2) and statistical analysis, including tests of normality. (XLSX). Note: *p*-values lower than 0,05 are highlighted in yellow.

**Supplementary Data File 4.** Effects of the blends on odorant receptor complexes expressed in oocytes from *Xenopus* *laevis.* Raw data was collected and normalized according to the blend or compound producing the greatest response (XLSX).

**Supplementary Table 1.** Primers used for qRT-PCR analysis (DOC).

**Supplementary Table 2.** List of compounds tested on oocytes from *Xenopus*. The compounds are organized into blends according to chemical class. Table includes supplier, compound purity, and CAS numbers (XLSX).

**Supplementary Table 3.** Raw and normalized data of dose response effects testing CpomOR22 to (*Z*)-6-undecenal (XLSX).

**Supplementary Figure 1.** Phylogeny of lepidopteran ORs. Canonical pheromone receptor clade is shaded in light blue. The highly supported clade including CpomOR22 is shaded in light grey. CpomOR22 is indicated with bold font and an asterisk. Likelihood ratio scores indicative of support greater than 70% are shown.

**Supplementary Figure 2.** Dose-response characteristics of CpomOR22-effects to (*Z*)-6-undecenal. The ab3A neurons expressing CpomOR22 generated spiking in response to the application of the compounds. The effects were concentration-dependent and reversible. Concentration dependences of the compounds are expressed as a function of normalized spike frequency [(Δspikes/s)/spikes] versus μg-doses. Responses were individually normalized to their saturating concentrations (50 µg). Error bars represent the standard error of the mean. Data were fit with the Hill equation (solid blue line). Below: spike trains elicited by doses ranging from 0.5 to 100.0 μg extracted from replicate 6 (Supplementary Table 3). Note: at saturating doses, we observed initial bleaching of the response effect. Red bar: stimulus (PDF).
